# Reduced gaze aftereffects are related to difficulties categorising gaze direction in children with autism

**DOI:** 10.1016/j.neuropsychologia.2013.03.021

**Published:** 2013-07

**Authors:** Elizabeth Pellicano, Gillian Rhodes, Andrew J. Calder

**Affiliations:** a Centre for Research in Autism and Education (CRAE), Department of Psychology and Human Development, Institute of Education, University of London, 25 Woburn Square, London WC1H 0AA UK; b ARC Centre of Excellence in Cognition and its Disorders, School of Psychology, University of Western Australia, Australia; c Medical Research Council, Cognition and Brain Sciences Unit, Cambridge, UK

**Keywords:** Autism, Gaze, Adaptation, Aftereffect, Vision

## Abstract

Perceptual mechanisms are generally flexible or “adaptive”, as evidenced by perceptual aftereffects: distortions that arise following exposure to a stimulus. We examined whether adaptive mechanisms for coding gaze direction are atypical in children diagnosed with an autism spectrum condition. Twenty-four typical children and 24 children with autism, of similar age and ability, were administered a developmentally sensitive eye-gaze adaptation task. In the pre-adaptation phase, children judged whether target faces showing subtle deviations in eye-gaze direction were looking leftwards, rightwards or straight-ahead. Next, children were adapted to faces gazing in one consistent direction (25° leftwards/rightwards) before categorising the direction of the target faces again. Children with autism showed difficulties in judging whether subtle deviations in gaze were directed to the left, right or straight-ahead relative to typical children. Although adaptation to leftward or rightward gaze resulted in reduced sensitivity to gaze on the adapted side for both groups, the aftereffect was significantly reduced in children with autism. Furthermore, the magnitude of children's gaze aftereffects was positively related to their ability to categorise gaze direction. These results show that the mechanisms coding gaze are less flexible in autism and offer a potential new explanation for these children's difficulties discriminating subtle deviations in gaze direction.

## Highlights

• Adaptive mechanisms are fundamental for perceptual coding.
• We found adaptation to gaze direction was significantly attenuated in autism.
• The degree of adaptation was also linked to children's gaze acuity.
• This study provides potential evidence for the functional benefits of adaptation.

## 1 Introduction

Detecting where someone is looking is immensely important for everyday social interactions. It is a skill that provides critical clues to another person's object of attention and their mental and emotional state. Infants are highly attuned to such cues very early on in development. They can detect and respond preferentially to eye contact at 3–4 months of age (Farroni, Mansfield, Lai, & Johnson, 2003) and, by the end of the first year, are able to understand the ostensive nature of gaze cues (Senju and Csibra, 2008). Such sensitivity continues to improve during childhood – both in terms of the accuracy with which one perceives gaze direction (Doherty, Anderson, and Howieson, 2009) and the use of gaze direction as an indicator of another's interest or attention (Baron-Cohen, Campbell, Karmiloff-Smith, Grant, & Walker, 1995). Together with other foundational abilities, sensitivity to gaze direction is held to play an important role in the development of understanding other minds (Baron-Cohen, 1995).

Given its prominence in theoretical accounts of social cognition, much attention has been devoted to understand the way that gaze direction is perceived and interpreted in autism – a heritable, lifelong neurodevelopmental condition that has its most striking effects on individuals' socio-communicative functioning. Atypical patterns of reciprocal gaze are a hallmark of autism (American Psychiatric Association (APA), 2000). Autistic^1^ children engage less in direct eye-to-eye contact (Sigman, Mundy, Sherman, & Ungerer, 1986) and tend not to monitor the target of another person's gaze in social contexts (Leekam, Hunnisett, & Moore, 1998). Furthermore, in gaze-reading tasks, autistic children do not use gaze information as a mentalistic cue (Baron-Cohen et al., 1995) and autistic adults show difficulties inferring another's mental state from the eye region (see Nation & Penny, 2008, for review).

Autistic people are not completely insensitive to gaze direction, however. Children with autism show basic knowledge about eyes and seeing (Tan and Harris, 1991), can detect whether someone is looking at them or not (Baron-Cohen et al., 1995) and shift their attention in gaze cueing tasks in a similar way to typical children (see Nation & Penny, 2008, for review). These findings have led some researchers to propose that there is a dissociation between detecting and interpreting gaze direction in autism (Baron-Cohen, 1995, Pelphrey et al., 2005), and that the real difficulty in autism lies not in discriminating gaze direction but in interpreting directional information from gaze during joint (triadic) interactions.

Yet several studies using more sensitive gaze categorisation tasks have found that the story might not be so straightforward. For example, Campbell et al. (2006) reported that school-age autistic chil-dren performed more poorly than typical children when asked to make fine-grained judgments about gaze direction (0°, 2°, 4° and 8° left/right). Similarly, school-age children, but not adolescents, with autism showed difficulties detecting the target of another's gaze when targets were separated by 5° and 10° (Webster and Potter, 2008; see also Riby & Doherty, 2009). Together, these findings suggest that the gaze discrimination mechanisms of individuals with autism might be less tuned to subtle deviations in gaze direction, potentially following an unusually protracted developmental trajectory.

The current study investigated a new aspect of gaze processing in autistic children by examining the ability of their gaze perception system to adapt (i.e., adjust) to recent context, specifically prolonged exposure to a particular gaze direction. Adaptation is ubiquitous in sensory systems occurring for both relatively simple stimulus attributes, including colour (Webster & Mollon, 1991), orientation (Gibson & Radner, 1937) and motion (Mather, Verstraten, & Anstis, 1998) and for more complex ones too, including faces (Rhodes and Leopold, 2011, Webster and Macleod, 2011). It is thought to have far-reaching conse-quences for our perceptual experience, including improving perceptual discrimination and maximising sensitivity to novel (unadapted) and potentially relevant information (Webster & MacLeod, 2011).

In light of the well-reported face-recognition difficulties in autism, Pellicano, Jeffery, Burr, and Rhodes (2007) investigated whether adaptation to facial identity might be atypical in autism. They used a task in which prolonged exposure to a particular face biases subsequent perception away from that face in the opposite direction (Leopold et al., 2001, Rhodes and Jeffery, 2006). Although both groups of children showed significant aftereffects, the extent to which autistic children shifted their perception following adaptation was significantly attenuated relative to typical children of similar age and ability. Pellicano and colleagues suggested that this diminished adaptation could reflect reduced updating or “tuning” of face norms with experience and that this may contribute to the face perception difficulties in autism.

Given the reported gaze perception atypicalities in autism, we were interested to determine whether gaze adaptation was similarly reduced in children with autism and whether the magnitude of their gaze aftereffects was significantly related to baseline performance in categorising small angles of gaze. Neuroimaging research has shown that dissociable neural systems underlie the perception and adaptation of facial identity and gaze, with identity associated with posterior ventral occipitotemporal regions and gaze the superior temporal sulcus (Haxby et al., 2000, Nummenmaa and Calder, 2009). Hence, attenuated gaze adaptation in autism would also suggest that the disrupted adaptation extends beyond the face recognition system to other aspects of face processing.

To test these hypotheses, we created a child-friendly version of a paradigm developed by Jenkins, Beaver, and Calder (2006). The original experiment showed that adaptation to faces with eyes averted 25° leftward resulted in a strong tendency to judge subsequent faces with leftward gaze as looking straight-ahead. Gaze averted to the right, unadapted side, however, remained unchanged or was more likely to be categorised correctly. A corresponding effect was found when adapting to 25° rightward gaze.

In the current study, children were first asked to judge whether faces showing subtle deviations in gaze were looking leftwards, rightwards or straight-ahead. Based on previous findings (Campbell et al., 2006, Webster and Potter, 2008), we expected that autistic children would be less accurate than typical children when making these fine-grained judgements about gaze direction. Children then adapted to faces gazing in one consistent direction (leftwards or rightwards) and their categorisation of gaze direction was re-assessed. Adaptation to leftward gaze, for example, should result in typical children incorrectly perceiving gaze averted to the left as looking straight-ahead, just as it does for typical adults (Calder et al., 2008, Jenkins et al., 2006). If attenuated adaptation to facial identity in autism (Pellicano et al., 2007) extends to other facial cues then we should expect children with autism to show smaller gaze aftereffects relative to typical children. Since autistic children are not entirely insensitive to gaze direction, we expected their gaze aftereffects to be reduced by a matter of degree rather than absent altogether. In addition, given the putative functional benefits of adaptation (cf. Clifford et al., 2007, Webster and Macleod, 2011), we predicted that the size of children's aftereffects would be positively related to their performance on the pre-adaptation gaze categorisation task.

## 2 Method

### 2.1 Participants

Forty-eight children aged between 9 and 14 years participated in this study. Twenty-four children (21 boys) diagnosed with an autism spectrum condition were recruited through the UK's National Autistic Society and community contacts in south west England, UK. All children had received an independent clinical diagnosis of either autism (*n*=19) or Asperger syndrome (*n*=5) according to DSM-IV (APA, 2000) criteria and further met criteria for an autism spectrum disorder on both the Lifetime version of the Social Communication Questionnaire (SCQ; Rutter, Bailey, & Lord, 2003; Table 1) and the Autism Diagnostic Observation Schedules – Generic (ADOS-G; Lord, Rutter, DiLavore, & Risi, 1999).

**Table 1:** Descriptive statistics for developmental variables for the autism and typical groups.

Group	Background variables *M* (SD) range			
	Chronological age years; months	Verbal ability^a^	Nonverbal ability^b^	SCQ score (out of 39)^c^
Autism	11; 2 (1; 5)	102.08 (12.00)	38.96 (7.10)	27.33 (4.87)
	9; 0–14; 4	80–126	26–50	18–35
Typical	10; 10 (1; 8)	104.42 (10.30)	37.08 (6.39)	4.75 (3.44)
	8; 2–14; 4	82–120	24–54	0–11

Note:

a British Picture Vocabulary Scale (2nd ed.) (Dunn et al., 1997).

b Raven's Standard Progressive Matrices (Raven, Court, and Raven, 1992).

c Elevated scores reflect increased symptomatology.

Twenty-four typically developing children (19 boys) were recruited from the local community. These children scored well below the cut-off score of 15 for autism on the SCQ (Table 1), indicating the absence of clinically-significant autistic symptoms in this group.

No child had received an additional medical (e.g., epilepsy) or neuro-developmental (e.g., attention deficit hyperactivity disorder) diagnosis, was in receipt of medication, or obtained standard scores <80 on tests of receptive vocabulary (British Picture Vocabulary Scale, 2nd edition, BPVS-II; Dunn, Dunn, Whetton, & Burley, 1997) or nonverbal reasoning (Raven's Standard Progressive Matrices; Raven, Court, and Court, 1992). All parents reported normal or corrected-to-normal vision in their children.

The autism and typical groups were well matched for chronological age, verbal ability, and nonverbal ability (all ps>0.34) (Table 1).

### 2.2 Stimuli

**Fig. 1:**
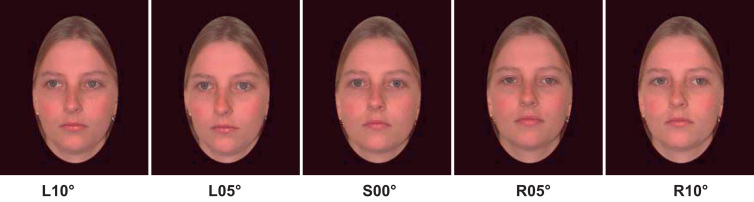
Example test stimuli displaying the five gaze angles (from the point-of-view of the observer) used in both the pre- and post-adaptation gaze discrimination tasks.

The experiment used a subset of Jenkins et al. (2006; Expt. 1) stimuli. The test and adapting stimuli consisted of colour photographs of the faces of 8 young adults (4 females) with neutral expressions. The test stimuli comprised the 8 models showing five angles of gaze – 10° left (L10), 5° left (L05), straight ahead (S00), 5° right (R05) and 10° right (R10) (Fig. 1). Images of the same 8 models displaying gaze averted 25° left and 25° right were used as adapting stimuli. The faces were presented in a black elliptical frame which masked most of their hair (Fig. 1). Test images subtended a visual angle of 16.4° (h)×10.2° (w) when viewed from a distance of 45 cm. The adapting images were 50% larger than test images to isolate the effects of high-level adaptation. The images were presented on a Macbook (15-inch LCD screen) using Psyscope software (Cohen, MacWhinney, Flatt, & Provost, 1993).

### 2.3 Procedure

To introduce children to the task, participants were shown two sets of images in succession, each with three images of a popular cartoon face. Of the three images in the first set, one displayed gaze averted 25° left, one showed gaze straight-ahead (0°) and one displayed gaze averted 25° right. The second set showed more subtle deviations of gaze (one example each of 10° left, 0° and 10° right). Following each presentation, children were asked to indicate the direction the person was looking (left, straight-ahead, or right; from the point-of-view of the observer) by pressing one of three colour-coded keys on the keyboard. Following correct answers to each question, children proceeded to the task proper, which consisted of four phases and lasted approximately 15–20 min.

#### 2.3.1 Pre-adaptation phase

**Fig. 2:**
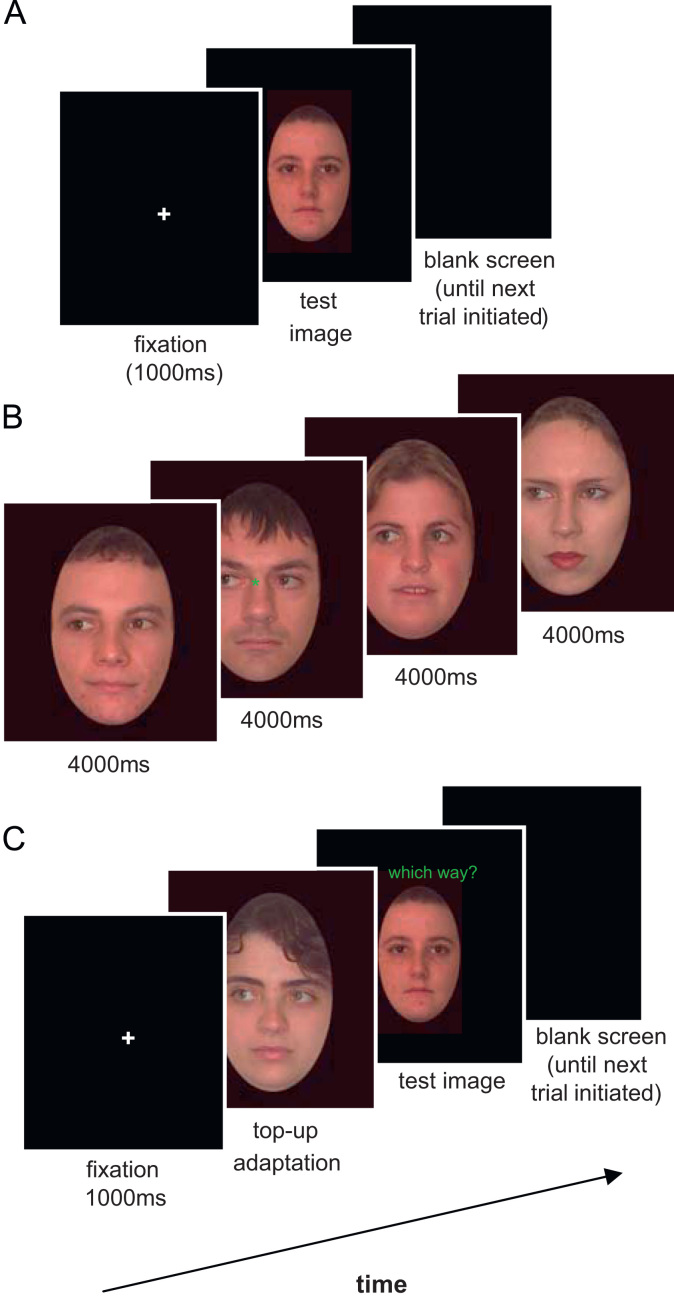
A summary of the events in each trial for (A) pre-adaptation, (B) adaptation and (C) post-adaptation phases.

This phase assessed participants' gaze acuity in the absence of adaptation. Each trial comprised a central fixation cross (1000 ms) followed by a centrally-presented test image (1500 ms) and a blank screen. Children were asked to decide (by making the appropriate key-press) whether the eyes were directed leftwards, straight-ahead or rightwards (Fig. 2A). There were 40 test trials in total (8 identities×5 gaze angles) presented in a randomized order. The onset of each trial was controlled by the experimenter to ensure that children's attention was focused on-screen. Twelve practice trials were administered at the outset of this phase to ensure that children understood task instructions. Feedback was given during practice but not test trials.

#### 2.3.2 Adaptation phase

During this phase, children observed a series of faces showing gaze averted in a single direction (leftwards 25° or rightwards 25°) for approximately 1.5 min. Each child saw 24 images in total (8 identities×3 presentations) for 4000 ms each presented in a randomized order (Fig. 2B). To ensure that they attended to the adapting stimuli, the children were asked to look carefully at the faces and to press the space bar when they detected a green star between the eyes, which appeared on one third of trials for a brief period (300 ms) randomly interspersed during the adaptation phase. In addition, the experimenter monitored the children's gaze to ensure that they fixated on the adapting faces for the full exposure duration.

#### 2.3.3 Post-adaptation phase

Immediately following the adaptation phase, participants completed a second gaze acuity task. Similar to the pre-adaptation phase, children saw 40 test faces for 1500 ms each, and were asked to indicate their gaze direction using the appropriate keys. During the post-adaptation phase, however, each test image was preceded by a 4-second “top-up” adapting image showing gaze in the adapted direction (25° left or 25° right) (Fig. 2C), which served to maintain adaptation. Pairs of test and top-up images were presented in a pseudo-random order with the condition that consecutive adapting and test images could never be of the same identity. The words “which way?” were clearly displayed above test faces to ensure that participants responded to the test image rather than the top-up adaptation image. The experimenter initiated the onset of each new trial. Following a short delay to ensure some decay of adaptation, the adaptation and post-adaptation phases were repeated with adaptation to the opposite gaze. Half of the participants adapted to leftward gaze first and half adapted to rightward gaze first.

#### 2.3.4 General procedure

Children were tested individually in a quiet room at either the University or the family home. Tests of receptive vocabulary (BPVS-II) and nonverbal reasoning (Raven's matrices) were always administered before the gaze adaptation task.

### 2.4 Analysis

**Fig. 3:**
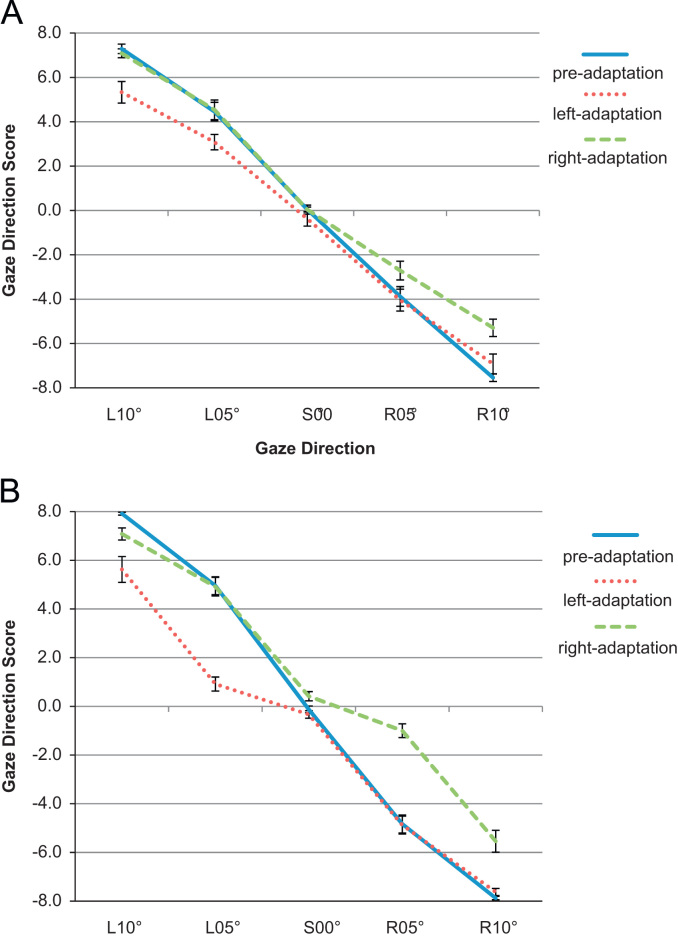
Graphs showing mean gaze direction score as a function of gaze angle during each adaptation condition for (A) children with autism and (B) typically developing children.

We calculated a “gaze direction score” to quantify children's bias to judge each gaze angle as directed leftwards, rightwards or straight-ahead by assigning each response the following values: left=1, straight-ahead=0, and right=−1 (see Teufel et al., 2009). These scores were then summed for each gaze angle for the pre-adaptation and post-adaptation phases. Positive values indicate a bias to judge gaze as left; negative values reflect a bias to judge gaze as right (Fig. 3).

Preliminary data analysis with order of presentation (left-adaptation first, right-adaptation first) entered as an additional factor in analysis of variance (ANOVA) revealed no main effect of order or any interaction involving order. Consequently, order of presentation was not included in subsequent analyses. Where appropriate Greenhouse-Geisser correction was used.

## 3 Results

The primary aims of this study were to establish whether autistic children showed diminished gaze adaptation compared to typical children and whether the magnitude of the aftereffect was associated with pre-adaptation performance in gaze categorisation.

The effects of adaptation were examined by performing a repeated-measures ANOVA on children's gaze direction scores with adaptation condition (left-adaptation, right-adaptation), gaze angle (L10, L05, S00, R05, R10) and group (autism, typical; between-participants) as factors (see Fig. 3). There were main effects of adaptation condition, *F*(1,46)=144.05, *p*<0.001, *η*_*p*_^2^=0.76, and gaze angle, *F*(1.89,184)=547.96, *p*<0.001, *η*_*p*_^2^=0.92. These were qualified by interactions between adaptation condition and gaze angle, *F*(3.06,184)=9.61, *p*<0.001, *η*_*p*_^2^=0.17, adaptation condition and group, F(1,46)=11.58, *p*=0.001, *η*_*p*_^2^=0.20, and a three-way adaptation condition×gaze angle×group interaction, F(4,184)=6.27, *p*<0.001, *η*_*p*_^2^=0.12. There was no main effect of group, F(1,46)=0.77, *p*=0.38, and no angle×group interaction, *F*(4,184)=1.58, *p*=0.18.

**Fig. 4:**
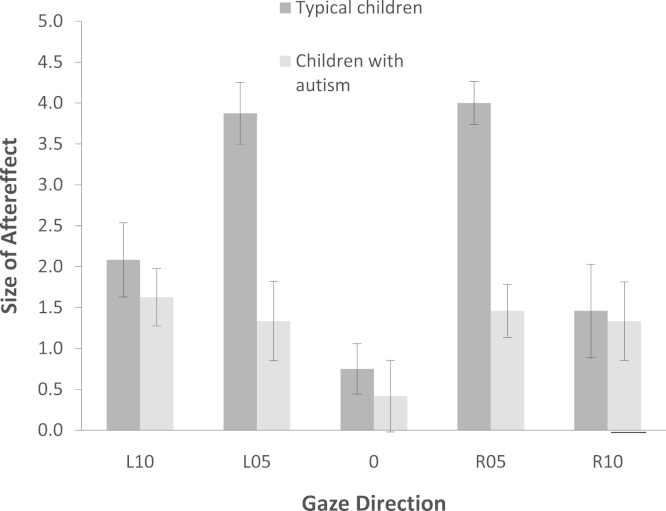
Graph shows the size of the aftereffect (i.e., the difference between the two adapting conditions) for each gaze angle as a function of group. Error bars show ±1 SEM.

To examine the source of the three-way interaction, we calculated the size of the aftereffect for each gaze angle as the difference between the two adapting conditions (i.e., gaze-direction score after right-adaptation minus the gaze-direction score after left-adaptation). Both groups of children showed significant aftereffects for R10, R05, L05, and L10, all *p*s<0.01, but not for straight-ahead, *p*s>0.08. Planned independent *t* tests on the difference scores revealed significant group differences for L05, *t*(46)=6.10, *p*<0.001, and R05, *t*(46)=4.14, *p*<0.001, only (all other *p*s>0.42), indicating that autistic children showed significantly *smaller* gaze aftereffects than typical children (Fig. 4).

Additional analyses compared children's pre-adaptation gaze-direction scores to their scores following left- or right-adaptation. Consistent with Jenkins et al. (2006; Expt. 1), the effects of adaptation were restricted to the adapted side in both groups of children (adapted side, ps<0.03; unadapted side: ps>0.09). Also like Jenkins et al., adaptation to gaze deviated either leftwards or rightwards had no significant effect on the perception of direct gaze (ps>0.13).

There were no significant group differences in children's ability to detect the stars during adaptation to leftwards (typical: *M*=7.6, SE=0.1; autism: *M*=7.5, SE=0.1), *F*<1, or rightwards gaze (typical: *M*=7.5, SE=0.1; autism: *M*=7.4, SE=0.2), *F*<1, suggesting that children in each group were attending to the adapting stimuli to a similar extent.

Table 2 shows children's accuracy scores during the pre-adaptation phase. To examine whether children with autism show difficulties perceiving subtle deviations in gaze direction, we performed a repeated-measures ANOVA on children's pre-adaptation accuracy scores with gaze angle and group as factors. There was a main effect of gaze angle, *F*(2.31,184)=87.83 *p*<0.001, *η*_*p*_^2^=0.66, reflecting significantly more accurate categorisation of gaze averted 10° (left and right) and direct gaze (0°) than gaze averted 5° (ps<0.001). There was also a main effect of group, *F*(1,46)=4.98, *p*=0.03, *η*_*p*_^2^=0.10, reflecting less accurate perception of gaze in autistic children (*M*=5.9; SE=0.2) relative to typical children (*M*=6.5; SE=0.1), but no gaze angle×group interaction, *F*(4,184)=1.39, *p*=0.24.

**Table 2:** Children's accuracy (number correct out of eight) in the pre-adaptation condition for each gaze direction.

**Condition**	**10° left***M* (SE)	**5° left***M* (SE)	**0° (direct)***M* (SE)	**5° right***M* (SE)	**10° right***M* (SE)
**Group**					
Autism	7.75 (0.14)	4.04 (0.44)	6.71 (0.13)	3.92 (0.46)	7.38 (0.18)
Typical	7.92 (0.06)	4.96 (0.37)	6.71 (0.09)	4.83 (0.37)	7.88 (0.07)

Correlational analyses between autistic children's overall baseline accuracy and the size of the aftereffect (averaged across gaze direction conditions) yielded a significant correlation, *r*(23)=0.41, *p*=0.02 (one-tailed), reflecting that smaller aftereffects were associated with poorer gaze categorisation. Typical children showed a borderline correlation, *r*(23)=0.30, *p*=0.08 (one-tailed).

## 4 Discussion

Gaze perception is vital for social interaction. Adaptation experiments with typical adults suggest that it recruits a multichannel, dynamic system, whereby the perceived direction is determined by relative activation of separate neuronal channels, tuned to (at least) three different gaze directions (left, direct, right) (Calder et al., 2007, Calder et al., 2008, Jenkins et al., 2006). The present study used a develop-mentally-appropriate aftereffect paradigm to investigate the extent to which gaze perception adapts in autistic and non-autistic children.

For typical children, adaptation to eyes averted 25° left or right resulted in an increased tendency to perceive gaze in the adapted direction as looking straight-ahead, whereas perception of gaze in the unadapted direction did not differ significantly from pre-adaptation performance. These data suggest that, as in adults (Jenkins et al., 2006, Calder et al., 2007), leftward and rightward gaze are coded by separate neural channels in children. Note that the adaptation and test faces used in our task were of different sizes to prevent retinotopic mapping, hence the aftereffects observed are likely to reflect high-level, directionally-specific encoding.

Children with autism also showed an effect of adaptation that was restricted to the adapted side, suggesting that a similar framework supports gaze perception in both typical and autistic individuals. Critically, however, the effects of adaptation in the children with autism were significantly reduced relative to typical children of similar age and ability, suggesting that dynamic calibration to other's gaze is attenuated in autism. It is interesting that children with autism showed reduced adaptation relative to typical children for gaze averted 5°, but not for gaze averted 10°. It is possible that this differ-ence reflects different tunings of the cells responsive to different gaze directions in the two groups. Testing this proposal, however, would require additional detailed experiments looking at a range of adapting directions and is beyond the scope of the current study.

Diminished adaptation to gaze direction is consistent with recent reports of reduced adaptation to facial identity in children with autism (Pellicano et al., 2007). Thus, the attenuated adapta-tion extends beyond the coding of facial identity to include another critical high-level facial attribute, gaze, that is supported by a separate neural system to facial identity (Haxby et al., 2000, Nummenmaa and Calder, 2009). An obvious question for future research is whether reduced adaptation in autism extends to non-facial or non-social stimuli, including lower-level perceptual properties, such as colour or orientation.

Children with autism were also significantly less accurate at categorising subtle deviations in gaze direction than typical children during the pre-adaptation phase. These findings are inconsistent with the view that individuals with autism show no difficulties per-ceiving gaze direction (Baron-Cohen, 1995) and instead corroborate reports of subtle atypicalities in their gaze perception (Campbell et al., 2006, Webster and Potter, 2008). The problems with gaze categorisation in this sample of autistic children, however, cannot explain their reduced adaptation since adaptation was measured by comparing the left and right adaptation conditions independent of baseline differences in gaze categorisation.

Because adaptation calibrates sensory systems according to the prevailing environment, it may have functional benefits, including improved discriminability around the adapted state and enhanced detection of novel inputs (Armann et al., 2011, Clifford et al., 2007, Kohn, 2007, Webster and Macleod, 2011). Although evidence for such benefits is equivocal (Webster & MacLeod, 2011), a number of positive findings have been reported (see Armann et al., 2011, for a discussion of work showing enhanced discrimination of faces). Nothing is known about the functional benefits of adaptation to gaze direction. Yet, the significant correlation between gaze categorisation accuracy and the magnitude of autistic children's aftereffects found here raises the possibility that less flexible gaze-coding mecha-nisms in autism might contribute to the apparently reduced fine-grained sensitivity to gaze direction.

Individuals with autism putatively show primary, early-emerging difficulties orienting towards social stimuli specifically (Klin, Jones, Schultz, Volkmar, & Cohen, 2002). There is also some evidence to suggest that autistic people look less at the eyes and more at the mouth relative to non-autistic people (Pelphrey et al., 2002, Dalton et al., 2005), although consistent evidence in favour of the latter has not been forthcoming (Falck-Ytter and von Hofsten, 2011). Nevertheless, could inattention to the face or eyes of the adapting faces potentially explain the group difference in gaze adaptation? Either form of inattention should lead to reduced adaptation for both 5° and 10° gaze directions yet, as already discussed, 10° left and right gaze showed equivalent adaptation in the two groups (ps>0.42). In addi-tion, the children were carefully monitored to ensure that they were concentrating during the adaptation task and there were no group differences on star-detection performance during adaptation trials. Inattention to the adapting stimuli is therefore an unlikely explanation of the reduced gaze adaptation, although the use of eye-tracking methodology would be necessary to completely rule out this explanation.

It is possible, however, that the subtle difficulties in gaze perception and reduced gaze adaptation reflect insufficient experience with faces during critical periods of the developing gaze perception system. Conversely, basic problems in adaptation might potentially cause some of the putative problems with visual inspection of faces. One idea is that adaptation helps to improve coding efficiency by dynamically adjusting the response properties of neurons to match the stimuli to which we are exposed (Clifford, 2002, Clifford et al., 2007). This calibration reduces the transmission of redundant information and maximises sensitivity to relevant novel information. Any problems continuously tuning one's experience to the current environment (i.e., adaptation) could therefore have knock-on effects for how efficiently one extracts, and attends to, relevant information from the environment, including gaze direction.

In recent years, adaptation has also been shown using neuroimaging methods, such as functional magnetic resonance imaging (fMRI-adaptation, a.k.a. repetition suppression). In one study, Calder et al. (2007) used fMRI-adaptation to show selective adaptation to left and right gaze in the right anterior superior temporal sulcus (STS) in typical adults. Separate work has shown that the posterior lateral fusiform gyrus and inferior occipital gyrus show adaptation to repetitions of the same facial identity (Andrews & Ewbank, 2004). On the basis of the current results and earlier behavioural work showing reduced facial identity adaptation in autistic children (Pellicano et al., 2007), it would be interesting to examine whether individuals with autism show less fMRI-adaptation to repetitions of the same gaze direction or same facial identity in the STS and posterior occipito-temporal cortex, respectively.

In summary, this study demonstrated reduced high-level gaze aftereffects in children with autism, suggesting that dynamic calibration of the gaze perception system is attenuated in these children. Moreover, the magnitude of these children's gaze aftereffects was significantly related to their pre-adaptation gaze categorisation. This finding offers a new explanation for difficulties discriminating subtle deviations in gaze direction in autism and future work should explore this link further (see Pellicano & Burr, 2012, for discussion). Our results also show that reduced adaptation in autism is not restricted to the face recognition system, but extends to another facial property, namely gaze direction, served by a separate neural system.

## Footnotes

1 The term “autistic individuals” is the preferred language of some people on the spectrum (see Sinclair, 1999). In this paper, we use this term as well as person-first language to respect the wishes of all individuals on the spectrum.
